# *Agrobacterium tumefaciens*-Mediated Transformation of *Valsa mali*: An Efficient Tool for Random Insertion Mutagenesis

**DOI:** 10.1155/2013/968432

**Published:** 2013-12-04

**Authors:** Caixia Wang, Xiangnan Guan, Hanyan Wang, Guifang Li, Xiangli Dong, Guoping Wang, Baohua Li

**Affiliations:** ^1^College of Agronomy and Plant Protection, Key Lab of Integrated Crop Pest Management of Shandong Province, Qingdao Agricultural University, 700 Changcheng Road, Qingdao 266109, China; ^2^Department of Molecular Genetics, The Ohio State University, Columbus, OH 43210, USA; ^3^College of Plant Science and Technology, Huazhong Agricultural University, 1 Shizishan Road, Wuhan 430070, China

## Abstract

*Valsa mali* is a causal agent of apple and pear trees canker disease, which is a destructive disease that causes serious economic losses in eastern Asia, especially in China. The lack of an efficient transformation system for *Valsa mali* retards its investigation, which poses difficulties to control the disease. In this research, a transformation system for this pathogen was established for the first time using *A. tumefaciens*-mediated transformation (ATMT), with the optimal transformation conditions as follows: 10^6^/mL conidia suspension, cocultivation temperature 22°C, cocultivation time 72 hours, and 200 **μ**M acetosyringone (AS) in the inductive medium. The average transformation efficiency was 1015.00 ± 37.35 transformants per 10^6^ recipient conidia. Thirty transformants were randomly selected for further confirmation and the results showed the presence of T-DNA in all hygromycin B resistant transformants and also revealed random and single gene integration with genetic stability. Compared with wild-type strain, those transformants exhibited various differences in morphology, conidia production, and conidia germination ability. In addition, pathogenicity assays revealed that 14 transformants had mitigated pathogenicity, while one had enhanced infection ability. The results suggest that ATMT of *V. mali* is a useful tool to gain novel insight into this economically important pathogen at molecular levels.

## 1. Introduction

*Valsa mali* (anamorph *Cytospora* sp.) is an important causal agent of apple and pear trees canker disease, which includes two varieties, *V. mali* var. *mali* occurring exclusively on apple and *V. mali* var. *pyri* occurring on both apple and pear [1, 2]. Valsa canker of apple and pear is the destructive disease that causes serious economic losses in eastern Asia, especially in China [3, 4]. The fungus causes elongated cankers on twigs, branches, and trunks. When cankers develop, they can girdle twigs, branches, and trunks, which then lead to desiccation and death of the distal part and even result in death of the entire tree. This disease poses severe threats to apple and pear production in China [4–6] and Japan [7, 8]. Currently, all apple varieties cultivated are very susceptible to the infection by *V. mali* with only some apple rootstocks being resistant [7, 9]. The incidence and severity of pear canker disease depend on pear varieties, with only *Pyrus ussuiensis* Maxim being resistant, but *P. bretschneideri* Rehd, *P. pyrifolia* (Burm.f) Nakai, *P. sinkiangensis* Yü, and *P. communis* L show susceptibility to *V. mali* var.* pyri* [9, 10]. Therefore, it is difficult to control Valsa canker disease of apple and pear by planting resistant varieties.

Up to now, the main disease management strategies for apple and pear canker disease include strict management of cultivation, chemical treatment, and scraping away diseased scars; however, these strategies are of no significance in controlling the disease [4]. The inefficiency of Valsa canker disease control mainly stems from the lack of understanding of the pathogenicity genes and their corresponding pathogenicity mechanisms at molecular levels of the causal agent [11]. Therefore, isolation of the genes involved in pathogenicity and characterization of their roles are crucial for elucidating fungal pathogenicity mechanisms, which can be approached by genetic mutagenesis analysis. Genetic approaches, generating a large mutant population by disruption or replacement of genes and then studying the related fungal phenotypes, are powerful tools for deciphering gene function [12–15]. Among many fungal genetic transformation systems, *A. tumefaciens*-mediated transformation is easy to operate, highly efficient, and widely applied to different recipients. In addition, transformants are genetically stable with mainly single copy T-DNA insertion in the fungal genomes [16, 17]. The efficiency of ATMT in fungi depends upon several factors, including fungal cell type and density, the *A. tumefaciens* strain, the time and temperature of cocultivation, and the concentration of the inducer (acetosyringone, AS) during co-cultivation [18–20].

Since the first application of ATMT on filamentous fungi by De Groot and colleagues [21], this technique is now widely used for the transformation of many fungi. Rho et al. [22] constructed a mutant library for *Magnaporthe oryzae* using ATMT and several infectious and pathogenic mutants were identified to be involved in appressorium formation, melanin synthesis, and cell wall degrading enzymes. Similarly, a T-DNA mutant library for *Setosphaeria turcica* was established by Wang et al. [23] via ATMT and several mutants were obtained related to the development and pathogenicity of this fungus. The establishment of a *V. mali* transformation system with high efficiency is a key to investigate the functions of this pathogen's genes and their corresponding pathogenicity mechanisms at molecular levels. This research presents an ATMT method for *V. mali*, which should serve as a useful tool for researchers working on this important pathogen. Colony morphology and conidia development and pathogenicity are several key criteria for the identification of mutants [24, 25]. Although the conventional PEG-based method was developed for *V. mali* var. *mali* transformation [11], it is laborious to prepare protoplast and the transformation efficiency is low for many fungi [26, 27].

In this study, *V. mali* var. *pyri* isolate LXS240101 was successfully transformed using ATMT method, and this procedure was optimized to increase transformation efficiency. Furthermore, T-DNA inserted transformants were screened and the biological properties as well as pathogenicity phenotypes of corresponding mutants were analyzed, the results of which indicated that ATMT is a suitable tool for insertional mutagenesis in *V. mali*.

## 2. Materials and Methods

### 2.1. Strains and Plasmids

The wild-type strain LXS240101 of *V. mali* var. *pyri* was isolated from the infected pear tree branches in Yantai Commercial Pear Orchard, a main pear producing area in Shandong province, China. During this study, this wild-type strain and transformants were stored and grown on potato dextrose agar (PDA) medium at 4°C and 25°C, respectively. *Agrobacterium tumefaciens* strain EHA105 [28] was stored in 20% glycerol solutions at −80°C until used. pBIG3C [29] containing hygromycin B resistant gene *hph* was kindly gifted by Professor Youliang Peng (China Agricultural University).

### 2.2. Primers and Media

Primers used for hygromycin gene PCR amplification were *hph*up, 5′-AAAGCCTGAACTCACCGCGACG-3′, *hph*down, 5′-CGGTTTCCACTATCGGCGAGTACTTCTAC-3′.

Yeast extract and peptone (YEP) medium were prepared as follows: Bactopeptone 10 g, yeast extract 5 g, NaCl 5 g, agar 15 g and adjust pH to 7.0 and the total volume to 1000 mL. PDA medium was made of 200 g peeled potatoes, glucose 20 g, agar 12 g; adjust the final volume to 1000 mL. Minimal medium (MM) included K-buffer 10 mL (200 g/L K_2_HPO_4_, 145 g/L KH_2_PO_4_, pH 7.0), M-N buffer 20 mL (30 g/L MgSO_4_·7H_2_O, 15 g/L NaCl), 20% glucose solution 10 mL and (w/v), 1% CaCl_2_·2H_2_O (w/v) 1 mL, 0.01% FeSO_4_ (w/v) 10 mL, and 20% NH_4_NO_3_ (w/v) 2.5 mL; adjust pH to 7.0 and the total volume to 1000 mL. Inductive medium (IM) included K-buffer (pH 4.9) 0.8 mL, M-N buffer 20 mL, and 1% CaCl_2_·2H_2_O (w/v) 1 mL, 20% glucose solution 10 mL (w/v), 20% NH_4_NO_3_ (w/v) 2.5 mL, 50% glycerol (v/v) 10 mL, 0.01% FeSO_4_ (w/v) 10 mL, 1 mg/mL 2-(N-morpholine) ethyl sulfonic acid sodium salt (MES) 10 mL; adjust the final volume to 1000 mL.

### 2.3. Antibiotics Sensitivity Assays of *A. tumefaciens* and *V. mali*

To test the growth inhibition of hygromycin B on *V. mali*, PDA plates were prepared, containing different hygromycin B concentrations with 0, 25, 50, 75, 100, and 150 *μ*g/mL. *V. mali* strain LXS240101 was first inoculated on fresh PDA medium without antibiotics. Three days later, inoculum was obtained from *V. mali* plates using 6 mm punch and inoculated into the center of the plates with different antibiotic concentrations. After cultivation at 25°C for seven days, the diameter of mycelium growth was measured to evaluate the growth inhibition. Four replicates were used during this assay. As with *A. tumefaciens*, bacteria solutions with an OD of 0.15 were spread on PDA plates with different concentrations of cefotaxime sodium as 0, 100, 200, 300, and 400 *μ*g/mL. After growing for three days at 28°C, the growth states of the colonies were evaluated.

### 2.4. Preparation of *V. mali* Conidia and *A. tumefaciens*

After growing for three days on PDA, *V. mali* strain LXS240101 was reinoculated onto new PDA plates and incubated at 25°C in the dark until the mycelium spread across the whole plate, and then it was transferred to 10°C for further cultivation in the dark. Three weeks later, massive white-color pycnidia were produced which reached maturity to release yellow conidia after five to seven days.

Plasmid pBIG3C was introduced into the *A. tumefaciens* strain EHA105 by electroporation at 2.5 kV and 25 *μ*F. The transformed EHA105 containing pBIG3C was spread on YEP plate (containing 50 *μ*g/mL kanamycin, 50 *μ*g/mL streptomycin, and 50 *μ*g/mL rifampicin). Two days later at 28°C, a single colony was inoculated into liquid MM medium supplemented with 50 *μ*g/mL kanamycin and cultivated at 28°C for 24 h at 180 rpm. Then the culture was diluted to OD_600_ of 0.15 in IM medium amended with different concentrations of AS (0, 100, 200, 300, and 400 *μ*M) and 1.0 mg/mL MES. Cells were grown under the same conditions to reach an OD_600_ of 0.6~0.8.

### 2.5. *A. tumefaciens*-Mediated Fungal Transformation and Transformants Screening

The *A. tumefaciens*-mediated fungal transformation was performed as described previously [30]. *V. mali* conidia were collected and suspended in sterile water and centrifuged at 2500 g/min for 10 min. After centrifugation, the supernatant was discarded. The *A. tumefaciens* prepared before was used to adjust the concentration of *V. mali* conidia to be 10^3^, 10^4^, 10^5^, 10^6^, and 10^7^/mL. Co-cultivation plates containing 200 *μ*M AS and 1.0 mg/mL MES were prepared with a piece of sterile cellophane covering the plates. 200 *μ*L mixture of *V. mali *spores and *A. tumefaciens* were spread on the co-cultivation plates and cultured for 0, 24, 48, 72, and 96 h at 22, 25, and 28°C, respectively. Later, the cellophane was transferred into a new Petri dish with the surface containing a mixture of *V. mali* conidia and *A. tumefaciens* facing up, upon which 20 mL PDA medium containing 50 *μ*g/mL hygromycin B and 300 *μ*g/mL cefotaxime sodium was placed. After three to five days at 25°C, single colonies were transferred onto new PDA plates (with 50 *μ*g/mL hygromycin B) for further cultivation. Colonies that could grow on the new PDA plates containing 50 *μ*g/mL hygromycin B were presumed as transformants.

### 2.6. Molecular Analysis of *V. mali* Transformants

Genomic DNA of transformants, as well as control (wild-type strain LXS240101), was extracted according to microwave method [31] and analyzed using PCR with *hph*up and *hph*down as primers to amplify *hph* gene. PCR reaction was carried out using 50 ng of total DNA, 4 *μ*M of primer, 0.2 mM of each dNTP, 10 × PCR buffer, and 1.5 units of Taq DNA polymerase (Takara Co. Ltd. Dalian, China). The PCR program was as follows: 94°C, 3 min; 94°C, 45 s; 61°C, 45 s; 72°C, 1 min for 35 cycles; 72°C, 5 min. The amplification products were separated by electrophoresis on a 1% agarose gel, stained with ethidium bromide and visualized under UV light.

Large scale DNA extraction from transformants and wild type was performed according to a procedure established by Stewart JR and Via [32]. After extraction, about 15 **µ**g genomic DNA was digested with *Hin*dIII (Takara Co., Ltd., Dalian, China). Digested DNA was separated on a 0.8% agarose gel, with digested wild-type DNA as negative control and digested pBIG3C (by *Apa*I, Takara Co. Ltd. Dalian, China) as positive control, which was then transferred to a Hybond N+ bond membrane (Roche Diagnostics, Basel, Switzerland). *hph* gene amplified from pBIG3C plasmid by PCR was used as probe and southern blot was carried out using DIG High Prime DNA Labeling and Detection Starter Kit (for color detection with NBT/BCIP) following the company's protocol (Roche Diagnostics, Basel, Switzerland).

### 2.7. Mitotic Stability of Transformants

To test whether the insertion was genetically stable or not, 30 randomly selected transformants of *V. mali* were cultured on PDA medium without hygromycin B at 25°C for seven days, from which the new mycelia were inoculated onto a fresh PDA plate in the absence of hygromycin B. After repeating this process for five generations, the mycelia were inoculated onto PDA plate containing 50 *μ*g/mL hygromycin B to check the growth of mycelium. This assay was repeated four times.

### 2.8. Assays for Biological Characters of Transformants

Wild-type strain LXS240101 and transformants with stable phenotypes were inoculated onto PDA plates without hygromycin B and cultivated for five days at 25°C to observe the colony morphology. Disks with 6 mm diameter were taken from the edge of the colonies (both wild-type strain and transformants) and placed onto PDA plates without antibiotics. These plates were put in the dark at 25°C and the colony diameters were recorded to evaluate growth rates. This experiment was repeated three times and each time four plates were used for corresponding strains.

Wild-type strain LXS240101 and transformants were induced to produce conidia that were collected using the same amount of sterile water. The spore morphology was recorded and the quantity of spores was counted. Then spore suspensions were diluted to a concentration of 10^5^/mL using PDB (PDA without agar) and left at 25°C in the dark to germinate. Twenty-four hours later, aniline blue solution was mounted to stop spore germination and samples were observed under microscope to record germination ratios. Every time around 200 conidia were observed to record germination ratios and this experiment was repeated for four times.

### 2.9. Assays for Pathogenicity of Transformants

Two-year-old pear twigs of *P. pyrifolia *(Burm. f.). cv. Nakai were collected from young trees planted at the experimental station of Qingdao Agricultural University. The inoculation method followed the procedure described by Suzaki et al. [33] with minor modifications described by Zhang et al. [34]. Twigs were cut into 30~40 cm long segments and washed with tap water, sterilized in 75% ethanol for 3 min, and then rinsed with sterile water for three times. The ends of the twigs were sealed with wax. Using an electric iron, four holes with a diameter of 1-2 mm were burnt into each twig segment; then 25 uL conidia suspension (10^6^/mL) or colony disks (diameter 6 mm) from either wild-type strain LXS240101 or *V. mali* transformants were placed into each hole. In total, 30 transformants (PFL1–PFL30) were used. Inoculated twigs were kept in crisper under 25°C and 100% relative humidity in the dark. Seven days later, lesion sizes were measured as indicators for disease development. Each twig had four inoculating points and five twigs were used for each strain. The pathogenicity assay was confirmed on *P. pyrifolia *(Burm. f.). cv. Nakai pears grown in the fields [35]. Inoculations were made on green shoots using a 6 mm cork borer to remove bark and by placing an agar plug from the growing margin of a 5-day-old colony directly on the fresh wound, which was covered with Vaseline and wrapped with Parafilm. Shoots were collected after eight weeks for laboratory examination. Fungi obtained from reisolation were identified based on colony morphology and PCR assay.

### 2.10. Data Analysis

SPSS software (Standard Version 16.0, SPSS Inc., Shanghai, China) was used to analyze the data, and all of the values are expressed as the mean ± the standard deviation (SD). A one-way analysis of variance (ANOVA) and least significance difference (LSD) tests were employed to evaluate the statistical significance (*P* < 0.05) of the results.

## 3. Results

### 3.1. Determination of Antibiotics Discriminating Concentration for *V. mali* and *A. tumefaciens*

**Figure 1:**
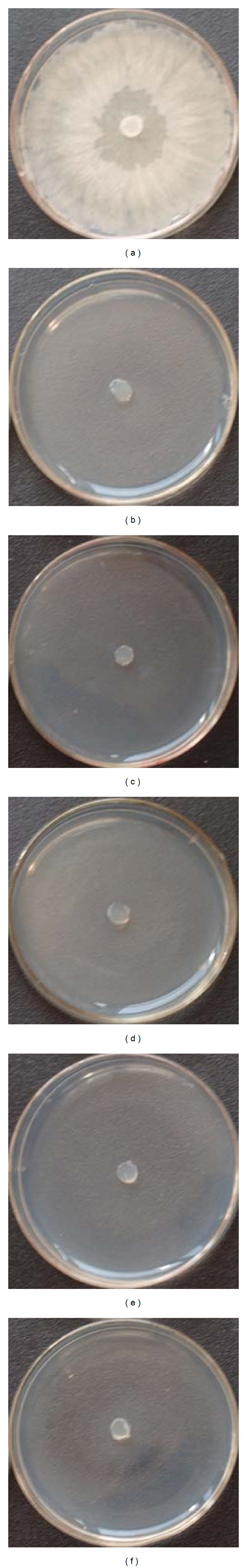
Growth of *Valsa mali* wild-type strain LXS240101 on PDA medium containing different concentrations of hygromycin B. LXS240101 shows normal growth on PDA plates without hygromycin B (a) and no growth on PDA plates containing 25, 50, 75, 100, and 150 ug/mL hygromycin B (b–f). The plates were incubated at 25°C for seven days.

Since later we wanted to use hygromycin B to identify transformants, we first tested the effects of hygromycin B on the growth of *V. mali*, wild-type strain LXS240101. Fresh *V. mali* disks were inoculated onto PDA plates containing different hygromycin B concentrations as 0, 25, 50, 75, 100, and 150 *μ*g/mL. Seven days later, this pathogen could grow normally on PDA plate without antibiotics, while its growth was completely inhibited by 50 *μ*g/mL hygromycin B. When growing on plates with 25 *μ*g/mL hygromycin B, *V. mali* hyphae growth was significantly inhibited, but still some conidia could germinate, as shown in Figure 1. Therefore, 50 *μ*g/mL hygromycin was used to select transformants.

*A. tumefaciens* EHA105 were grown on PDA plates with various concentrations of cefotaxime sodium. The results showed that agrobacteria grew well on PDA plates with 100 *μ*g/mL cefotaxime sodium, but the growth was inhibited by 200 *μ*g/mL cefotaxime sodium, with only 10 to 15 colonies showing up. However, agrobacteria failed to grow when 300 *μ*g/mL cefotaxime sodium was applied. Therefore, 300 *μ*g/mL cefotaxime sodium was used to prevent agrobacteria growth after cocultivating *V. mali* and *A. tumefaciens.*

### 3.2. Establishment and Optimization of *V. mali* Transformation

**Figure 2:**
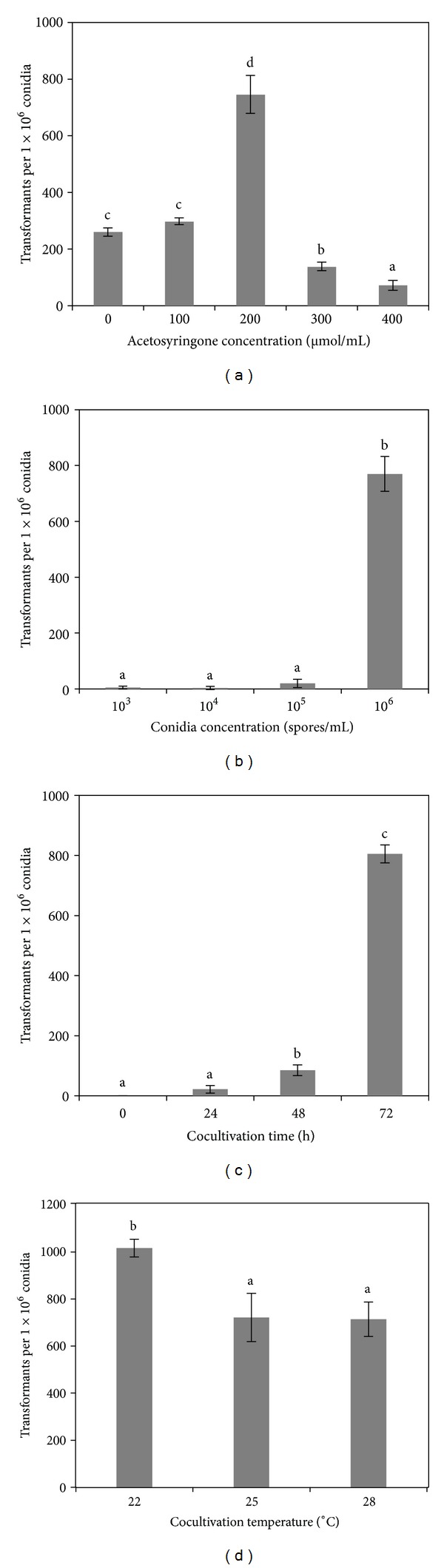
The effects of acetosyringone concentration (a), conidia concentration (b), co-cultivation time (c), and co-cultivation temperature (d) on the transformation efficiency of *Valsa mali* strain LXS240101. These four factors exhibit obvious impacts on the transformation efficiency. Data are the mean ± SD of triplicate experiments. Different letters on the bar in the same histogram show statistical significance (*P* < 0.05) on the transformation efficiency.

The transformation of *V. mali* is mediated by *A. tumefaciens* harboring the targeted plasmid. Four factors that may influence the transformation efficiency were taken into account and optimized, including AS concentration, recipient conidia concentration, co-cultivation temperature, and co-cultivation time. The effects of AS on transformation efficiency were tested by adding to the IM different concentrations of AS as 0, 100, 200, 300, and 400 *μ*M, as shown in Figure 2(a). With 100 *μ*M AS, the number of transformants per 10^6^ conidia achieved was similar to that obtained with no AS in the medium. Transformation efficiency increased with rising concentrations of AS and peaked at 200 *μ*M AS in the medium with 745.00 ± 67.27 transformants identified per 10^6^ conidia. However, fewer transformants were achieved when the AS concentration was further increased, with only 71.67 ± 14.33 transformants per 10^6^ conidia at 400 *μ*M. Therefore, AS concentration has obvious impacts on transformation efficiency and 200 *μ*M is the optimal concentration for *V. mali* transformation.

To test the effects of recipient conidia concentration on transformation efficiency, conidia with different concentrations, 10^3^/mL, 10^4^/mL, 10^5^/mL, 10^6^/mL, and 10^7^/mL, were mixed with the same concentrations of *A. tumefaciens*, respectively, and cocultivated for three days (Figure 2(b)). With higher concentration of recipient conidia, more transformants were identified. Specifically, when the concentration was between 10^3^/mL and 10^5^/mL, the transformation efficiency was similar and low (*P* < 0.05). However, the average transformation efficiency was 770.00 ± 62.45 transformants per 10^6^ conidia when using 10^6^/mL recipient conidia and it kept increasing as higher concentration of recipient conidia was used, but the calculation was rendered less accurate due to the overlap of transformant colonies. Therefore, the concentration of recipient conidia could impact the transformation efficiency.

Conidia *A. tumefaciens* mixtures were placed on co-cultivation medium and cultivated at 25°C and dark conditions. Transformation efficiency was tested for co-cultivation times as 0 h, 24 h, 48 h, 72 h as well as 96 h, respectively. As shown in Figure 2(c), no transformants were achieved when cocultivated for 0 h using PDA medium containing hygromycin B and cefotaxime. Longer time of co-cultivation promoted transformation and 805.00 ± 30.00 transformants per 10^6^ conidia were screened when co-cultivated for 72 h. 96 h co-cultivation time rendered too many colonies overlapping with each other, and thus the counting was inaccurate.

22°C, 25°C, and 28°C were used to test the effect of co-cultivation temperature on transformation (Figure 2(d)). We identified similar numbers of transformants (720.00 ± 102.59 and 713.33 ± 73.20, resp.) per 10^6^ conidia under 25°C and 28°C, whereas 1015.00 ± 37.35 transformants were achieved per 10^6^ conidia under 22°C. Therefore, the optimal transformation conditions for *V. mali *include 200 *μ*M AS in IM-medium, 10^6^/mL recipient conidia, 72 h co-cultivation time, and 22°C co-cultivation temperature.

### 3.3. Confirmation of *hph* Gene Integration into the Fungal Genome

**Figure 3:**
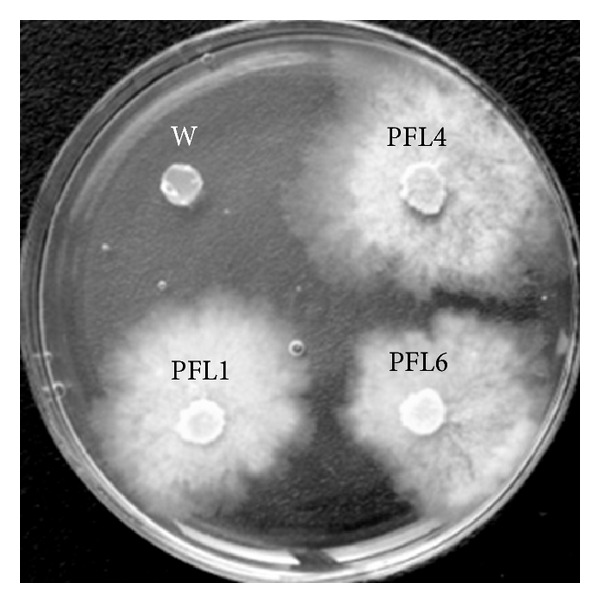
Genetic stability analysis of transformants on PDA medium containing 50 ug/mL hygromycin B after subculturing for five generations on PDA plates without antibiotics. Three independent transformants (PFL1, PFL4, and PFL6) exhibit normal growth on hygromycin B containing medium and wild-type strain LXS240101 (W) shows no growth. The plates were incubated at 25°C for 48 hours.

An assessment of the mitotic stability of transformants was carried out. Since several transformation experiments yielded around 1000 transformants in total, thirty transformants were randomly selected and inoculated onto PDA medium without antibiotics and subcultured for five generations. Then these transformants were inoculated onto PDA plates with hygromycin B to check their growth ability. The results showed that all the 30 transformants maintained their hygromycin B resistance after being subcultured for five generations in the absence of hygromycin B (Figure 3). The thirty transformants were selected and designated as PFL1–PFL30.

**Figure 4:**
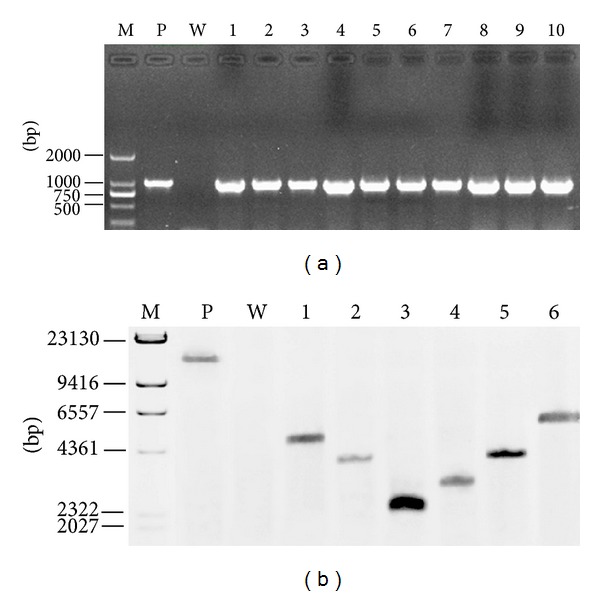
Molecular analysis of T-DNA integration into the genome of randomly chosen transformants resistant to hygromycin B. (a) Polymerase chain reaction assay with primers specific for the amplification of an internal 987 bp fragment of the* hph* gene. Lane P positive control with vector pBIG3C, W wild-type *Valsa mali* LXS240101 genomic DNA, Lanes 1–10 genomic DNA isolated from putative transformants PFL1–PFL10, and M DNA molecular weight markers with bases indicated on the left. (b) Southern blot analysis of transformants with DIG-labeled *hph* gene probe. P positive control with vector pBIG3C linearized with *Apa*I, W wild-type *Valsa mali* LXS240101 genomic DNA digested with *Hin*dIII, 1–6 genomic DNA isolated from putative transformants PFL1-PFL6 digested with *Hin*dIII, and M DNA molecular weight markers with bases indicated on the left. These six transformants show single copy inserts and the insertion sites are different.

Genomic DNA from the thirty selected transformants was isolated with microwave methods and PCR was performed using *hph*up and *hph*down primers with pBIG3C as positive control and the DNA from wild-type strain (LXS240101) as negative control. As shown in Figure 4(a), an *hph* specific fragment (987 bp) was detected in the pBIG3C control and in the transformants as well but was absent in the wild-type sample. The expected 987 bp PCR products were confirmed by sequencing. Therefore, PCR results confirmed that T-DNA was inserted into the genome of *V. mali.*

Large scale DNA isolation was applied to obtain DNA from six randomly selected transformants (PFL1–PFL6). Isolated DNA was subject to restriction endonuclease digestion by *Hind*III (site not present inside T-DNA), and Southern blot was performed with wild-type strain DNA as negative control and linearized pBIG3C (*Apa*I digested) as positive control. *hph* gene amplified from pBIG3C was used as probe. The hybridization yielded only a single band for each transformant and the positive control, but no signal was detected in the negative control (Figure 4(b)), indicating that T-DNA was successfully integrated into a single site of *V. mali* genome for these six transformants. Moreover, the presence of different sized bands in the Southern blot revealed random integration of the T-DNA of the majority of the transformants.

### 3.4. Biological Properties for Transformants

**Figure 5:**
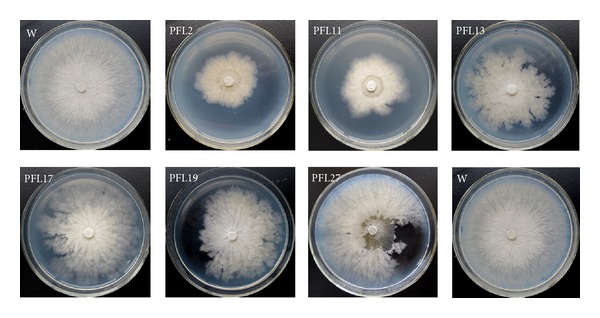
Colony morphology of six transformants and wild-type strain LXS240101 (W) on PDA medium. Wild-type LXS240101 (W) shows normal growth on PDA plates and 6 independent transformants (PFL2, PFL11, PFL13, PFL17, PFL19, and PFL27) exhibit different colonial morphologies and radial growth rates. The plates were incubated at 25°C for 72 hours.

Thirty transformants were recorded for morphology. Compared with wild-type strain LXS240101, transformants PFL2, PFL11, PFL13, PFL17, PFL19, and PFL27 showed abnormal morphology including deformation and fan-shaped colonies (Figure 5), while the rest had no obvious morphology changes. These transformants were also subject to recording growth rates (Table 1). The results revealed that 30.00% of the transformants showed significantly reduced growth rates, including PFL2, PFL3, PFL6, PFL11, PFL13, PFL17, PFL19, PFL23, and PFL30. Table 1 also showed the conidia productivity and germination ratios. Among those 30 transformants, PFL1, PFL13, PFL15, and PFL17 failed to produce conidia when growing on PDA medium. The rest 26 transformants could produce conidia with PFL2, PFL6, and PFL11 producing less conidia and another 6 transformants (PFL5, PFL9, PFL12, PFL23, PFL25, and PFL27) having significantly increased sporulation quantity when compared with wild-type strain LXS240101 (11.50 ± 2.06 × 10^6^ spores/plate). The amount of conidia produced by transformant PFL6 was only 0.13 ± 0.28 × 10^6^ spores per plate, while transformant PFL27 produced 387.50 ± 68.89 × 10^6^ spores per plate. The other 16 strains had similar conidia productivity to that of wild-type strain LXS240101. In addition, the conidia produced by the transformants had no difference from those of LXS240101 in morphology as well as in color. However, in terms of germination ratio, 92.18 ± 1.34% of the spores from LXS240101 could germinate, but 10 transformants produced spores with significantly reduced germination ratios and specially PFL-6 produced nongerminating spores.

### 3.5. Pathogenicity Assay for Transformants

**Figure 6:**
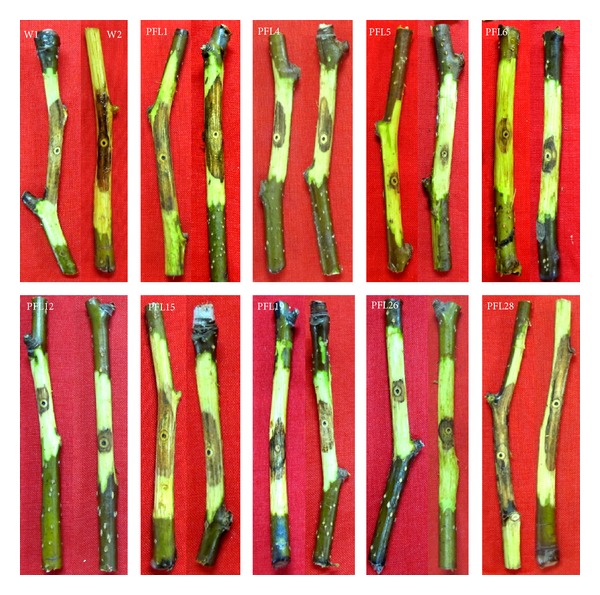
Lesions caused by *Valsa mali* wild-type strain LXS240101 and transformants in *Pyrus pyrifolia* (Burm. f.). cv. Nakai seven days after inoculation on two-year-old twigs using conidia suspensions or mycelium plugs. W1, PFL4, PFL5, PFL12, PFL19, PFL26, and PFL28 were inoculated using 100 uL conidia suspensions (10^6^/mL). W2, PFL1, PFL6, and PFL15 were inoculated using mycelium plugs (diameter 6 mm). The lesions caused by the transformants were different in size.

Pathogenicity assay was conducted using the conidia from wild-type strain and the 25 transformants that produced conidia with germinating ability. Spore suspensions were inoculated onto the wounds created by the electronic iron on the detached twigs. The other five transformants that produced no conidia or non-germinating conidia were tested using a mycelium plug (Figure 6 and Table 1). Compared with wild-type strain, which caused 14.66 ± 3.54 and 13.87 ± 2.98 cm^2^ lesion areas using conidia suspensions and mycelium plug, respectively, 15 transformants (50.00%) showed altered pathogenicity phenotypes including 14 transformants that produced small lesions and PFL18 that showed enhanced infection with the lesion area reaching 23.18 ± 0.80 cm^2^. Among the 14 transformants with reduced pathogenicity, PFL5, PFL9, PFL10, PFL12, PFL13, and PFL30 caused lesion areas that were less than 2 cm^2^. The pathogen was later reisolated from some of these lesions on selective medium (PDA containing 50 *μ*g/mL hygromycin B) and their identity [36] as well as the presence of the *hph* gene was confirmed by PCR assays. These 15 transformants reisolated from respective lesions were inoculated on pear green shoots in the field using mycelium plugs as inocula, and seven transformants were confirmed as low-pathogenicity mutants with slight or no mycelial formation and no development of visible lesions. The other seven transformants were medium-pathogenicity mutants with moderate mycelial formation and disease lesions that were significantly smaller than those caused by wild-type strain LXS240101. The transformant PFL18 was a high-pathogenicity mutant with massive mycelial formation and very large lesion areas (data not shown).

## 4. Discussion

Genetic transformation is a powerful tool to study gene functions of fungi and currently three main methods are in use for fungal transformation including PEG-mediated protoplast transformation, restriction enzyme mediated integration (REMI) transformation, and ATMT transformation [37–40]. It is laborious to prepare protoplasts for the PEG method and the transformation is inefficient, whereas REMI results in a high proportion of off-targeted fragment insertion or multiple insertions that pose difficulties to downstream analysis for many fungi [27, 41]. In contrast, *A. tumefaciens*-mediated transformation overcomes those shortcomings. This is a first report of successful ATMT of *V. mali* which is a very important economic pathogen on apple and pear trees in East Asia. This protocol offers various advantages compared to the PEG-mediated transformation method reported for *V. mali*. 1015.00 ± 37.35 transformants per 10^6^ conidia were obtained via ATMT, which are much more than the 44 transformants per 1 *μ*g DNA achieved with PEG method reported for this pathogen [11]. Moreover, ATMT could utilize conidia, instead of only protoplasts, for transformation. Protoplast preparation is usually expensive and laborious and also the lytic enzymes often have batch variations [42]. In addition to *V. mali* var. *pyri* strain LXS240101 transformation, we also applied this new method to transform another variety of this pathogen, *V. mali* var. *mali* (strain LXS080601 isolated from *Malus domestica* “Fuji”), and obtained around 605 transformants per 10^6^ conidia. At least 30 transformants tested for strain LXS080601 showed stable hygromycin B resistance (experiment not further shown). These results demonstrated that the ATMT technique is an efficient tool for *V. mali* var.* pyri* and *V. mali* var.* mali* transformations. In a following work, we will attempt to construct a large scale insertion library and on the basis of the biological properties and pathogenicity analysis, transformants will be selected to be subject to TAIL-PCR for isolation of targeted genes [12, 29]. These studies will be of critical importance in elucidating gene functions related to the development or pathogenicity of pathogen and laying foundation for pathogenesis characterization.

Although *A. tumefaciens*-mediated transformation is widely applied to filamentous fungi, its efficiency varies among different fungi and even the vectors or *A. tumefaciens* strains used could also affect its efficiency [16, 43]. In order to establish an optimal transformation system, binary vector pBIG3C and *A. tumefaciens* strain EHA105 were selected [29, 44], in addition to which, the recipient conidia concentration, co-cultivation time and temperature, and AS concentration in IM medium were also optimized. It was found that using 10^7^/mL recipient conidia and 96 h as co-cultivation time would result in the highest transformation efficiency; however, under those conditions, the number of putative transformants growing in selection plates was too high and it was difficult to isolate discrete colonies. AS is necessary for *A. tumefaciens*-mediated transformation, which is also validated by our results in which adding an optimum amount of AS (200 *μ*M) into inductive medium enormously enhanced transformation efficiency. However, too high a concentration of AS hindered the transformation, probably due to the toxic effects of high AS concentration posed on the recipient conidia, for example, inhibiting the germination of the conidia.

The research into the infectious processes for a pathogen is normally complicated by a series of genes involved in pathogenicity. The production of conidia is required for the pathogenicity of many pathogens and their numbers and germination ratios could also impact pathogenicity [19, 45]. Discovery of pathogenicity genes in *V. mali* will enable further understanding of infection mechanism of this important pathogen. T-DNA-tagged mutants with altered pathogenicity provide an attractive approach for the identification of the genes [42]. To analyze the biological properties and pathogenicity phenotypes in a collection of nearly 1000* V. mali* transformants, 30 were randomly selected and used. The biological properties including colony morphology, growth rate of mycelium, sporulation quantity, and germination ratio of conidia were detected *in vitro*. As with *V. mali* transformants, it was easy to uncover mutants associated with fungal development. Four sporulation deficient mutants were obtained on PDA medium, and they failed to produce conidia on inoculated pear twigs (data not shown). Six transformants significantly increased sporulation quantity; however, five of them had lower conidia germination ratio than wild-type strain. In pathogenicity bioassays on detached pear twigs, 14 transformants caused small lesions and one transformant caused large lesions. The results were achieved when using green shoots in the field as materials. These results clearly demonstrate the value of the ATMT methodologies in investigating the molecular mechanisms involved in *V. mali* infection process by characterizing the biological properties as well as pathogenicity bioassays of transformants.

## 5. Conclusion

Here we report, for the first time, the development and optimization of an *A. tumefaciens*-mediated transformation system for *V. mali*. This protocol enabled us to obtain a large number of T-DNA insertional transformants within a short experimental period and all the selected transformants displayed mitotic stability. Therefore, ATMT can be utilized as an efficient tool for insertional mutagenesis and further investigations into the molecular mechanisms of *V. mali* pathogenesis. This method has the potential to be adapted to transform other important fruit tree pathogenic fungi.

## Figures and Tables

**Table 1:** Comparison of some important biological properties and pathogenicity between transformants and wild-type strain LXS240101.

Strain	Colony diameter (cm)	Sporulation quantity (×10^6^ spores/plate)	Spore germination ratio (%)	Lesion areas (cm^2^)
LXS240101	8.78 ± 0.45	11.50 ± 2.06	92.18 ± 1.34	14.66 ± 3.54/13.87 ± 2.98^a^
PFL1	7.86 ± 0.67	0.00 ± 0.00*	—	13.15 ± 3.40
PFL2	3.99 ± 0.68*	0.75 ± 0.36*	94.95 ± 1.91	5.54 ± 1.97*
PFL3	6.56 ± 0.26*	12.06 ± 3.42	86.52 ± 8.96	11.99 ± 1.89
PFL4	8.84 ± 0.11	16.94 ± 4.05	88.82 ± 9.33	3.47 ± 0.58*
PFL5	8.03 ± 0.36	240.00 ± 35.18**	59.22 ± 9.82*	1.23 ± 0.07*
PFL6	5.62 ± 0.16*	0.13 ± 0.28*	0.00 ± 0.00*	2.65 ± 0.43*
PFL7	8.84 ± 0.39	11.25 ± 4.74	81.93 ± 11.13	8.93 ± 4. 18
PFL8	8.27 ± 0.35	23.75 ± 11.57	81.73 ± 10.42	8.01 ± 2.27
PFL9	8.31 ± 0.99	116.88 ± 22.49**	22.93 ± 5.23*	1.64 ± 0.67*
PFL10	8.18 ± 0.34	15.00 ± 5.14	86.92 ± 7.33	1.43 ± 0.56*
PFL11	4.27 ± 0.25*	2.75 ± 0.81*	75.02 ± 14.48	13.02 ± 2.30
PFL12	8.24 ± 0.54	150.00 ± 60.11**	51.88 ± 9.75*	1.35 ± 0.65*
PFL13	5.79 ± 0.25*	0.00 ± 0.00*	—	1.79 ± 0.43*
PFL14	8.23 ± 0.13	37.50 ± 13.87	83.62 ± 6.82	8.30 ± 1. 80
PFL15	8.58 ± 0.48	0.00 ± 0.00*	—	10.24 ± 0.91
PFL16	8.71 ± 0.35	20.63 ± 9.22	13.32 ± 4.54*	2.21 ± 0.46*
PFL17	7.28 ± 0.56*	0.00 ± 0.00*	—	4.70 ± 1.53*
PFL18	9.00 ± 0.00	26.31 ± 5.65	90.54 ± 3.43	23.18 ± 0.80**
PFL19	6.63 ± 0.22*	23.44 ± 14.04	17.49 ± 9.29*	4.94 ± 0.38*
PFL20	8.26 ± 0.35	15.94 ± 2.59	92.10 ± 2.49	13.35 ± 0.91
PFL21	8.16 ± 0.57	25.00 ± 4.74	9.88 ± 1.10*	4.98 ± 0.11*
PFL22	7.83 ± 0.78	18.75 ± 2.05	85.57 ± 5.26	12.57 ± 3.55
PFL23	6.69 ± 0.22*	156.88 ± 40.47**	85.82 ± 4.12	7.82 ± 4.49
PFL24	8.30 ± 0.36	7.50 ± 3.56	85.15 ± 11.19	10.39 ± 1.25
PFL25	7.94 ± 0.54	110.63 ± 24.27**	57.82 ± 9.01*	8.17 ± 2.29
PFL26	8.24 ± 0.17	9.50 ± 2.97	94.87 ± 3.78	3.63 ± 1.32*
PFL27	8.05 ± 0.42	387.50 ± 68.89**	69.95 ± 13.33*	4.98 ± 0.98*
PFL28	8.54 ± 0.28	13.56 ± 3.11	88.66 ± 9.03	11.11 ± 0.47
PFL29	8.72 ± 0.07	34.38 ± 12.62	62.00 ± 14.03*	7.18 ± 3.98
PFL30	7.36 ± 0.11*	17.13 ± 4.13	89.34 ± 5.28	1.88 ± 0.38*

Duncan's multiple range test between wild-type strain LXS240101 and its transformants, respectively; ^a^indicated lesion areas caused by conidia suspensions/mycelium plug; *indicated significantly decreased values for transformants at *P*_0.05_; **indicated significantly increased values for transformants; —: indicated that the trial was not conducted.
